# Chlorate of Potash Internally in Ulcerated Stomatitis, Either Mercurial or Not

**Published:** 1855-08

**Authors:** 


Chlorate of Potash Internally in Ulcerated Stomatitis, either Mercurial or not.—It would appear, from experiments made by M. Herpin, of Geneva, and M. Blache, of Paris, that chlorate of potash has a wonderful efficacy in the above affections. The salt is given in doses of from half a drachm to a drachm in some mucilaginous vehicle; this is given three or four times in the twenty-four hours, and a palpable improvement is at once observed. The *topical* use of this solution has not been found so efficacious.—*Ibid.*

